# Artificial intelligence challenges in the face of biological threats: emerging catastrophic risks for public health

**DOI:** 10.3389/frai.2024.1382356

**Published:** 2024-05-10

**Authors:** Renan Chaves de Lima, Lucas Sinclair, Ricardo Megger, Magno Alessandro Guedes Maciel, Pedro Fernando da Costa Vasconcelos, Juarez Antônio Simões Quaresma

**Affiliations:** ^1^GA.IA—AI Integrated Analysis Group, Remote Group, Brazil; ^2^Postgraduate Program in Tropical Diseases, Tropical Medicine Center, Federal University of Pará, Belém, PA, Brazil; ^3^MBA Program in Artificial Intelligence for Business, Faculdade Exame, São Paulo, SP, Brazil; ^4^Department of Pathology, State University of Pará, Belém, PA, Brazil; ^5^School of Medicine, São Paulo University, São Paulo, SP, Brazil

**Keywords:** artificial intelligence, biological threats, catastrophic risks, public health, safety

## Abstract

The threat landscape of biological hazards with the evolution of AI presents challenges. While AI promises innovative solutions, concerns arise about its misuse in the creation of biological weapons. The convergence of AI and genetic editing raises questions about biosecurity, potentially accelerating the development of dangerous pathogens. The mapping conducted highlights the critical intersection between AI and biological threats, underscoring emerging risks in the criminal manipulation of pathogens. Technological advancement in biology requires preventative and regulatory measures. Expert recommendations emphasize the need for solid regulations and responsibility of creators, demanding a proactive, ethical approach and governance to ensure global safety.

## Introduction

In the contemporary global scenario, biological threats represent a field of growing concern, notable for their capacity to trigger significant harm to human health, biodiversity, and socioeconomic stability. This context intertwines with the accelerated advancement of Artificial Intelligence (AI), a transformative tool that permeates various spheres of society. The intersection of these two domains promises not only to offer innovative solutions but also raises pertinent concerns about misuse and the amplification of risks associated with these challenges. In this context, it is crucial to explore the increasing impact of AI in these scenarios of biological threats, considering not only its positive contributions but also the emerging challenges and risks that this technological advancement might unleash.

## Perspective on catastrophic risks

In the book “The Precipice: Existential Risk and the Future of Humanity,” Toby Ord uses the term “precipice” as a metaphor for the delicate current state of humanity, perched on the brink of facing existential catastrophes with an imminent probability of occurrence at 16.67%. Ord discusses existential risks, which are threats to the long-term potential of humanity, potentially resulting in its extinction, such as a large asteroid impacting Earth. The realization of these risks would constitute what Ord terms an existential catastrophe. He argues that these risks are of utmost importance under various moral theories but criticizes humanity’s significant neglect of these dangers, as illustrated by the inadequate budget dedicated to the prohibition of biological weapons compared to the expenditure of an average fast-food franchise. The categories of risks include natural ones (the asteroid threat), which are less threatening due to advancements in mitigation, and anthropogenic and future ones, such as climate change, misaligned artificial intelligence, and planned pandemics, presenting significant challenges due to our inadequate preparedness (Namdar and Pölzler, 2021).

In this context, it is important to emphasize that impacts considered catastrophic at the national level may not hold the same severity on a global scale, and the same applies to the impact on different species within an ecosystem.

According to the World Health Organization, biological weapons are microorganisms such as viruses, bacteria, or fungi, or toxic substances produced by living organisms that are deliberately released to cause disease and death in humans, animals, or plants. These threats form a subset of a larger class of weapons, sometimes referred to as weapons of mass destruction, which also include chemical, nuclear, and radiological weapons. Among them, the use of biological agents is a critical concern, and it is thought that the risk of using these agents in a terrorist attack is increasing (World Health Organization, 2023).

Artificial intelligence (AI) typically refers to the capability of machines to simulate advanced intelligences. In the biological field, AI proves to be invaluable, especially with its algorithms capable of managing large volumes of unstructured data. This capability allows for rapid analyses and complex decisions, driving innovations across various sectors, such as the biosciences (Bhardwaj et al., 2022). However, this same capability also presents significant risks of malicious use, such as in the creation of dangerous biotechnologies.

It is already quite clear that AI has the potential to revolutionize several aspects of our lives, including how we respond to biological threats. However, there are emerging risks associated with the misuse of AI. AI could be used to develop new bioweapons or to enhance the effectiveness of existing ones.

As AI advancements propel progress, they can also enable its misuse as biological weapons. Sandbrink (2023) differentiates two classes of AI tools that could pose such biosecurity risks: Large Language Models (LLMs) and Biological Design Tools (BDTs). According to the author, LLMs can democratize access to biological knowledge, lowering barriers for its misuse. The combination of LLMs with BDTs could drastically expand both the capability and accessibility to biologically manipulate agents, increasing the risks of malicious actions. Although currently LLMs may generate inaccurate information due to certain limitations and still produce what is called “hallucinations,” which could frustrate potential malicious actors, their future evolution promises greater accuracy and influence in bioengineering.

The ability of LLMs to access and analyze vast amounts of information can create gaps in government regulation, allowing the emergence of risks associated with the misuse of AI to plan biological attacks. Preliminary results presented highlight the capacity of LLMs to provide guidance that, while not generating direct instructions for the creation of biological weapons, present relevant insights that could assist in the execution of these attacks (Mouton et al., 2023).

Advances in synthetic biology and multimodal AI (beyond the use of LLMs alone) can amplify the risk of the deliberate release of harmful viruses, enabling future AI-assisted systems to provide guidance from the selection of viral genomes to the synthesis and release of the virus, using multimodal training data including lecture videos and laboratory demonstrations. For example, they could create a supervirus combining the rapid spread of measles, the mortality rate of smallpox, and/or the incubation period of HIV. Strategies to balance the use of AI in synthetic biology, manage access to genetic information, and guide the development of AI capabilities and the use of synthetic tools become crucial in the mitigation process for future threats. This requires a careful risk–benefit analysis in gain-of-function research, considering the advances in synthetic biology techniques that can be enhanced by the use of AI to enable bioterrorism (Newman, 2024).

Insights like these illustrate the emerging challenges associated with the advancement of AI in the context of biological threats. While the preliminary results have not indicated the explicit generation of instructions for biological weapons, they demonstrate the capacity of LLMs to discuss fictional scenarios and identify potential pathogenic agents.

A paper prepared by researchers at the Center for AI Safety (Hendrycks et al., 2023), an organization with the mission of promoting the reduction of social-scale risks from AI, suggests that catastrophic AI risks can be grouped into four main categories: malicious use, AI race, organizational risks, and rogue AI. As for malicious use, AI could be used in bioterrorism to create new pandemics, for example. In the AI race, conflicts may spiral out of control with autonomous weapons and cyber warfare enabled by AI. As for organizational risks, organizations developing advanced AI could cause catastrophic accidents, especially if they prioritize profits over safety. And as for rogue AI, there is the risk of losing control over AI as they become autonomous. The AI could seek power and resist shutdown.

Biological threats represent a complex and diverse spectrum of dangers to humanity. According to Tyshenko (2007), emerging technologies such as genetic manipulation allow for the development of extremely lethal pathogens as biological weapons. Historically, disease outbreaks caused by natural pathogens or by accidents in laboratories and deliberate actions have devastated human populations. Furthermore, although the use of biological weapons is considered by experts to be a low probability event, the possibility exists and could lead to catastrophic global consequences.

Historical examples of global threats that originated from the emergence of natural pathogens include the Black Plague, the Spanish Flu, bioterrorism with anthrax, and, more recently, COVID-19. Therefore, given the destructive potential of these threats and their direct impacts on human health and global socioeconomic destabilization, the urgency to understand their origins, prevent malicious manipulation of biotechnological technology, and strengthen response strategies to mitigate such threats is highlighted.

According to West and Gronvall (2020), the advancement of CRISPR as an accessible and easy-to-use genetic editing tool brought not only numerous benefits for medical research but also raised serious biosecurity concerns. Originating in advanced research laboratories such as those at the University of California—Berkeley and MIT, CRISPR has expanded its use to various contexts, from university laboratories to do-it-yourself (DIY) initiatives worldwide. This democratization has increased concern over its possible misuse for the creation of biological weapons.

Artificial Intelligence, in addition to the CRISPR tool, can inadvertently be employed in the development of biological weapons if not properly directed toward ethical purposes. Its application in genomic analysis can potentiate the creation of more effective pathogenic variants, allowing the rapid manipulation of organisms to make them more harmful. AI algorithms have the potential to optimize genetic research, allowing faster identification of genes of interest. In a negative scenario, this could include genes related to virulence or resistance to treatments, facilitating the creation of more dangerous pathogens and potentially enhancing the development of biological weapons by malicious actors.

According to Hoffmann et al. (2023), advances in DNA synthesis and the increasing accessibility of synthetic genomic technology are challenging current biosecurity models, demanding urgent regulatory updates. The discussion on genetic biocontainment systems highlights the pressing need for control mechanisms to prevent the inadvertent dissemination of pathogenic agents.

The challenges faced by AI amidst emerging biological threats require a multifaceted and proactive approach. The convergence of AI and biology necessitates a thorough consideration of risks, especially considering the increased ability to create synthetic organisms.

An example that illustrates how genetic editing technology can be beneficially used for public health and global well-being is the release of genetically modified malaria mosquitoes in Burkina Faso (Yao et al., 2022). This pioneering study was crucial for understanding the fitness costs associated with transgenes and obtaining valuable information about the dynamics of these altered mosquitoes. These data are essential for the development of genetic control strategies, offering the promise of potentially significantly reducing the spread of malaria.

However, the AI that could potentially drive these advances also raises serious concerns. If not properly regulated, AI could be employed by malicious groups to manipulate genes in a way that creates resistant or more harmful mosquitoes, amplifying the challenges in combating malaria and potentially creating new biological threats.

A recent mapping conducted by GA.IA—Group for Integrated AI Analysis, a volunteer group of professionals committed to identifying, assessing, and predicting catastrophic risks associated with advanced AI models, including those that reach Artificial General Intelligence (AGI) capabilities, is presented in Table 1. The information revealed highlights the critical intersection between biological threats and AI. This detailed analysis emphasizes the emerging risks resulting from the convergence of criminal dissemination of biological manipulations and the potential role of AI in enhancing these pathogens for catastrophic purposes.

**Table 1:** Emerging risks at the intersection of biothreats and AI.

Emerging risk	Catastrophic damages	Amplification by AI
Criminal dissemination of genetically modified organisms (GMOs) as biological weapons affecting human health and biodiversity.	Widespread impacts with chaos and collapse of health systems, socioeconomic destabilization (travel restrictions, quarantines, border closures, and suspension of commercial activities), and even large-scale loss of lives. The dissemination of these modified pathogens can result in diseases for which there is no known treatment, spreading rapidly and challenging the capacity of health systems to respond adequately. This will cause panic and lead to significant social disruptions, mass health problems, elevated medical expenses, and negative impacts on the local economy.	AI can be used in advanced genetic research, which could be exploited to enhance the genetic engineering of these pathogens and use algorithms for protein remodeling, making them more lethal, resistant to treatments, or capable of adapting more quickly to different environments, complicating containment and the development of treatments.
Use of genetically programmed nanobots to attack specific organs or specific systems of the human body.	Rapid and widespread organic failure and massive collapse of health systems. Nanobots, if designed to attack vital organs or specific systems of the human body, could lead to rapid deterioration of essential tissues and bodily functions.	Using advanced machine learning algorithms, AI can process large sets of biomedical information, such as genomic data and cellular information. AI identifies specific patterns in cells, allowing the creation of nanobots capable of recognizing precise cellular targets. Through computational simulations and molecular modeling, AI designs nanobots with highly specialized functionalities. AI can employ immune system recognition strategies, identifying effective camouflage mechanisms for nanobots. AI analyzes data from existing medical treatments, identifying patterns that nanobots can bypass or resist.
Development of pathogens that attack the basic structures of the human genetic code.	Progressive and unpredictable degradation of human DNA, resulting in uncontrollable hereditary diseases, genetic deformations, mass sterility and an increase in cancer cases. This can lead to a drastic population decline, affecting socioeconomic dynamics.	AI can precisely map specific regions of the human genome that are susceptible to harmful modifications. This could lead to the creation of highly specialized pathogens capable of targeting and modifying the genetic code in a more effective and destructive way, generating irreversible health conditions.
Modifying microorganisms to attack agricultural crops and critical systems such as water supplies.	Widespread food shortages, hunger, increased conflicts over resources, mass migrations. and geopolitical instability. Damage to critical systems, collapse of vital infrastructure. and disruption of essential services.	AI can be applied to analyze plant genomes and identify specific vulnerabilities that, when attacked by GMOs, would lead to catastrophic failures. Furthermore, machine-learning algorithms can be applied to improve dissemination strategies for these microorganisms, aiming for rapid global spread. AI can map vulnerabilities in critical infrastructure, determining the most susceptible entry points. Algorithms can be developed to alter monitoring data, masking the presence of microorganisms and the extent of contamination.
Spread of “human control viruses.”	Researchers define “neuroweapons” (neurotechnological weapons) as technologies that can be used to influence or control neurological functions in order to achieve advantages in security and defense contexts, and discuss how neuroweapons can be of great interest to national security efforts, given the potential of these technologies to fundamentally alter the nature of war and espionage (Giordano and Wurzman, 2011). Mass psychological manipulation, behavior control, social division. and political destabilization. Malicious individuals could develop microorganisms that affect brain chemistry, leading to mass behavioral changes, inducing fear, paranoia, or even driving specific actions resulting in social and political chaos.	Using neuroscientific and psychological data, AI can understand the chemical processes and patterns of brain activity associated with emotions, behaviors and reactions. AI can analyze patterns and identify how microorganisms could affect these brain chemical processes. Machine learning algorithms can identify propagation channels such as water and air to ensure that microorganisms reach large numbers of people.
Bioweapons targeting specific ethnic groups and development of “signed” microorganisms.	Genetic disparities among ethnic groups suggest the possibility of developing biological weapons that target specific populations. Chemical agents could be engineered to exploit innate genetic vulnerabilities, enabling offensive operations that protect the aggressor’s population while incapacitating the target population. This approach raises significant ethical concerns about the use of bioweapons in future conflicts (Larson, 1970). The targeted use of GMOs can result in the mass death of specific ethnic groups, leading to genocide and cultural annihilation. The genetic complexity of “signature” microorganisms can make it difficult to identify the source of the attack, making it challenging to hold perpetrators accountable. Such attacks can destabilize not only specific regions but also entire nations, generating large-scale humanitarian and political crises. Attacks targeting ethnic groups can cause widespread distrust between nations, generating geopolitical tensions.	AI can assist in advanced genetic research. Bad actors could use AI to modify microorganisms to include specific “genetic signatures,” making them capable of attacking target populations while remaining undetectable by traditional tracking and identification methods.

As we explore AI-amplified biothreats, these risks can be categorized as hypothetical, emerging, and immediate: nanobots and human control viruses, for example, represent hypothetical risks with currently low probabilities, due to the need for future advancements in nanotechnology; the criminal dissemination of GMOs is an emerging risk with moderate probability, highlighted by advanced genetic engineering; and the modification of microorganisms to attack crops and critical systems constitutes an immediate and high-probability threat.

The criminal dissemination of genetically modified organisms and the development of pathogens that attack the basic structures of human genetic code as biological weapons can be classified as emerging, as there are significant capabilities in genetic engineering that could be misused, making this threat more imminent than hypothetical, but still dependent on further developments to reach the catastrophic levels described. And bioweapons targeted at specific ethnic groups and the development of “signed” microorganisms can also be classified as emerging, as the concept of targeted genetic weapons is plausible with current genetic knowledge, making this a threat that requires continuous monitoring.

The rapid technological evolution amplifies the potential for misuse of biology, increasing the risks of widespread harm. Scientific advances demonstrate that the detection of genetic modifications in organisms and the ability to identify the possible responsible laboratory from their genetic sequences, if developed, could become a powerful forensic tool for attributing outbreaks caused by genetically manipulated pathogens, offering defense against potential abuses of synthetic biology (Lewis et al., 2020).

Artificial Intelligence can be instrumental in the early detection of signs of genetic engineering, providing advanced analytical tools to identify subtle patterns indicating genetic manipulation. However, the accuracy and reliability of these detection systems must be prioritized, as erroneous attribution can have serious implications.

It is extremely necessary for governments, society, educational and research institutions to engage in discussions about the ethical dilemmas involving the use of AI to deal with such significant threats. The primary focus should be, but not limited to, the need for robust regulations and policies to govern the responsible use of AI in these situations.

Given the urgency of confronting biological threats, international collaboration is essential in creating safe and effective AI systems as a form of mitigation. By prioritizing ethics in the application of AI to mitigate threats, it should be ensured that technological benefits are accompanied by responsibility and safety.

Researchers propose strategies to avoid Global Catastrophic Biological Risks (GCBRs)—large-scale biological events that can cause severe harm to human civilization, potentially putting its long-term survival at risk—focusing on inhibiting the use of biological weapons by states and influential actors. These strategies include: (1) greater transparency and compliance in the Biological and Toxin Weapons Convention (BWC), (2) improved capabilities to identify the origin of biological events, and (3) a clear system of accountability for violations of the BWC (Yassif et al., 2023).

Strategies like these also highlight the interdependence with the risks associated with AI. While the focus is on preventing the development and use of biological weapons by states, the growing ability of AI to analyze and interpret biological data can play a significant role in the early detection and attribution of origins of events, or even to contribute to the potential amplification of risks. The enhanced transparency proposed in the context of the BWC could be complemented by transparency in the use of AI in biological analyses, strengthening the ability to identify potential threats.

One proposed form of mitigation could be the reliable identification of revealing signatures characteristic of different genetic designers, termed “genetic engineering attribution,” which aims to prevent misuse (Alley et al., 2020). In this pioneering work, the deteRNNt algorithm was developed, which, through an approach based on DNA motifs, phenotypic information, and recurrent neural networks (RNNs), achieved a lab attribution accuracy of over 70%. This advancement established a crucial milestone in identifying genetic signatures, paving the way for possible mitigation of the misuse of biotechnology.

Technologies like deteRNNt are vital against biological threats but bring challenges. Regulating their application is crucial for ethical use. Future development requires collaboration between bioengineering, AI specialist teams, and policy formulation.

Proactive recommendations provide an essential roadmap for the mitigation and prevention of catastrophic risks stemming from the advancement of AI. Crucial measures are recommended such as improving biosecurity, holding creators accountable for damages, safely regulating, publicly controlling AI, conducting audits, and restricting its use in high-risk environments without proven safety (Hendrycks et al., 2023).

## Discussion

In the challenging context of biological threats, the implications of AI emerge as a double-edged sword, offering the potential both to protect and to amplify risks. A collaborative and integrated approach is crucial, as the genetic enhancement of natural enemies, the production, and release of biological agents require multidisciplinary strategies. Environmental impact studies, essential in the face of releases of genetically manipulated organisms, become a key point in understanding global ramifications. Exhaustive safety tests are imperative to assess risks to human and environmental health with the misuse of AI.

Projecting into the future, a proactive approach is vital: understanding the operational risks of AI in this context will guide research toward robust solutions that protect global security from catastrophic risks in society. The intersection between AI and biological threats is a complex field, requiring not just technological innovation but also ethical consideration and careful governance to mitigate potential risks as rapid advancement continues.

## Data availability statement

The original contributions presented in the study are included in the article/supplementary material; further inquiries can be directed to the corresponding author.

## Author contributions

RL: Conceptualization, Formal analysis, Investigation, Writing – original draft. LS: Formal analysis, Writing – review & editing. RM: Formal analysis, Writing – review & editing. MM: Conceptualization, Formal analysis, Supervision, Writing – review & editing. PV: Conceptualization, Writing – review & editing. JQ: Conceptualization, Supervision, Writing – review & editing.
